# Right ventricular metastasis of leiomyosarcoma

**DOI:** 10.1186/1476-7120-7-20

**Published:** 2009-05-05

**Authors:** Magnus Dencker, Sven Valind, Martin Stagmo

**Affiliations:** 1 Department of Clinical Physiology and Nuclear Medicine, Malmö University Hospital, Lund University, Malmö, Sweden; 2 Department of Cardiology, Malmö University Hospital, Lund University, Malmö, Sweden

## Abstract

Metastatic presentation of leiomyosarcoma in the heart is very rare. We present transthoracic echocardiography and combined PET/CT images of a case with a large right ventricular metastasis of leiomyosarcoma. The patient was placed on cytostatic drugs for palliative purposes, but passed away one month later because of an untreatable ventricular tackycardia.

## Introduction

The word sarcoma is derived from the Greek word fleshy substance, and this group of neoplasm constitutes a very heterogeneous group [1]. Leiomyosarcoma of the uterus is a rather uncommon tumor, accounts for only a few percent of all cancers of the uterus, and is associated with poor prognosis [1,2]. Leiomyosarcoma primarily tend to metastasize to the peritoneal cavity, lung, lymph nodes, or liver [3]. Metastatic manifestations of leiomyosarcoma to the heart are very rare. There are only sporadic case reports of imaging in cases with leiomyosarcoma metastasis to the heart, and none include images from echocardiography and PET/CT [4-8]. The present case report includes transthoracic echocardiography and combined PET/CT images of a large right ventricular metastasis of leiomyosarcoma.

## Case presentation

**Figure 1:**
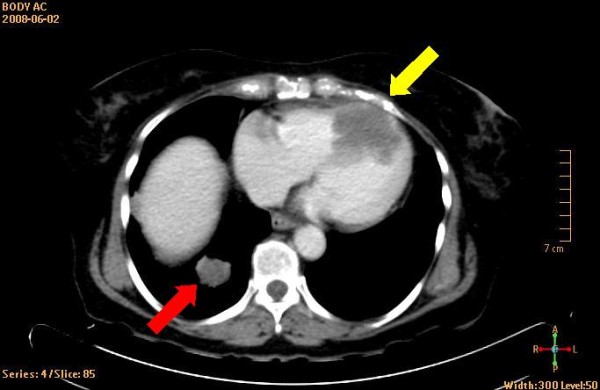
**Display of CT-image**. The yellow arrow indicates the metastasis of the leiomyosarcoma. The red arrow indicates of the lung metastases.

**Figure 2:**
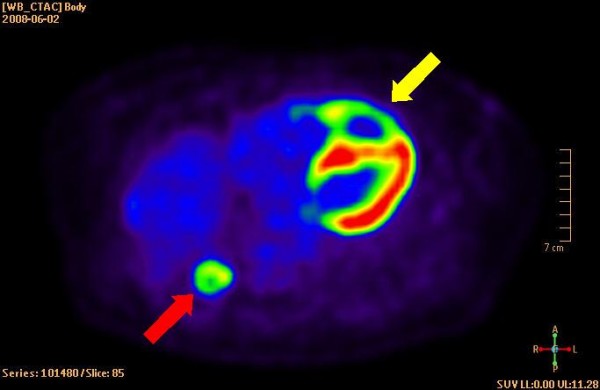
**Display of PET-image**. The yellow arrow indicates the metastasis of the leiomyosarcoma. Note the absence of FDG uptake in the center of the tumor, thus suggesting necrosis. The red arrow indicates of the lung metastases.

**Figure 3:**
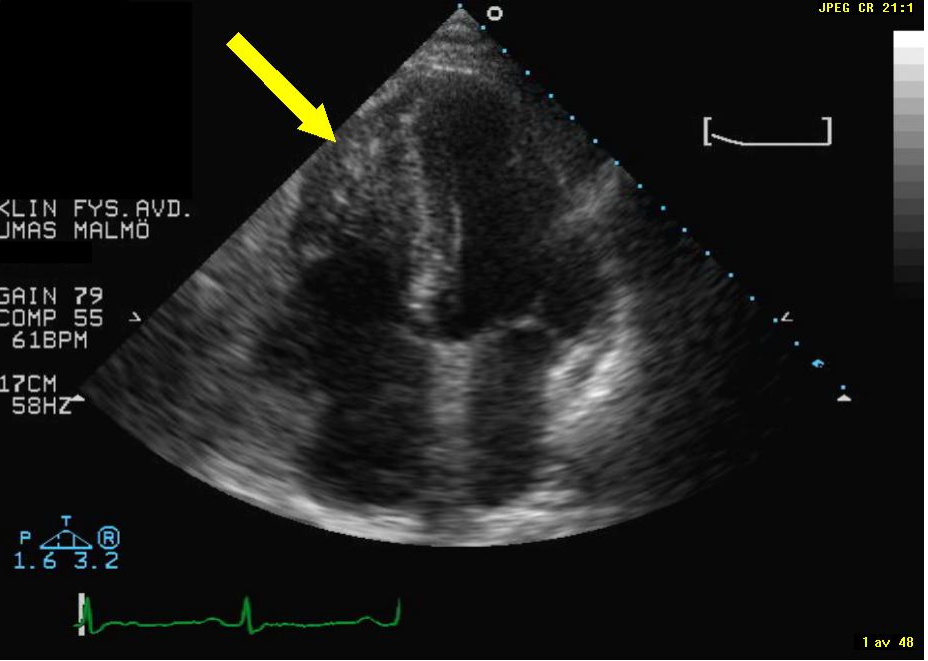
**Display of echocardiography image from apical four-chamber view**. The yellow arrow indicates the metastasis of the leiomyosarcoma.

The patient was a 69-year-old woman who 3-years earlier had undergone surgical resection of a leiomyosarcoma located in the corpus uteri. A recent chest X-ray had showed a tumor-suspected structure in the left lung. This was shown by fine needle biopsy to be a metastatic tumor of leiomyosarcoma. For primary tumor staging the patient underwent a combined computed positron emission tomography (PET) and computer tomography (CT) examination by an integrated PET/CT system (Gemini TF, Philips Medical Systems, Best, the Netherlands), after injection of ^18^F-fluorodeoxyglucose (FDG) [2]. Figure 1 displays a CT image that shows the rather large tumor located in the right ventricle and appears to engage the interventricularseptum. Figure 2 shows the corresponding PET image. Additional metastases were also found in both lungs. Standard transthoracic echocardiography examination was performed, as part of a cardiac evaluation, with a Sonos 5500 (Philips Medical Systems, Andover, Massachusetts, USA). A predominantly echodense structure was visualised in the apical portion of the right ventricle, from an apical four-chamber view (Figure 3 and Additional file 1). The structure measured approximately 5 × 3 centimetres and was occupying the apical half of the right ventricular cavity. The contraction of the interventricularseptum was significantly reduced presumably due to tumor growth into the interventricularseptum (Additional files 1, 2 &3). Stroke volume measurement by Doppler indicated a stroke volume from the right ventricle of 86 ml. The patient was placed on cytostatic drugs for palliative purposes, but passed away one month later because of an untreatable ventricular tackycardia.

## Discussion

The present case report includes transthoracic echocardiography and combined PET/CT images of a rare case with a large right ventricular metastasis of leiomyosarcoma. Metastatic manifestations of a leiomyosarcoma to the heart are associated with very poor prognosis and our patient passed away only one month later because of an untreatable ventricular tackycardia. The use of a radiopharmaceutical agent such as FDG provides the capability for imaging tumor glucose metabolism, whereas CT images provide anatomical information [9]. The CT images indicated that the tumor was growing through the septum. This could, however, be due to motion artifacts since the image acquisition was not ECG-gated. The image of FDG uptake indicated, for a malignant tumor, moderately elevated uptake in the rim of the tumor, as expected for a leiomyosarcoma [2], whereas the centre of the tumor displayed no uptake thus suggesting necrosis. Furthermore, there was a significant reduction of glucose uptake in the septum, compared to the lateral wall, which was probably due to tumor growth. This hypothesis was supported by the fact that the contraction of the interventricularseptum was significantly reduced, presumably due to the tumor growth into the interventricularseptum. It is also of interest that the cardiac output of the right ventricle was preserved at rest in our case, despite the fact that almost 50% of the right ventricular lumen was obliterated by the metastasis of the leiomyosarcoma.

The echocardiography diagnosis of a tumor within the heart is not always straightforward [10]. Differential diagnosis that should be considered, apart from metastasis, include primary cardiac tumor -primarily myxoma, vegetation, or thrombus. Assessing the location of the mass combined with echogenicity of the mass, and naturally including patient history often result in a accurate diagnosis. While it has been suggested that contrast echocardiography improves the diagnostic capacity of transthoracic echocardiography examination with respect to differentiating intracardiac masses [11,12], the evidence for this is scarce. The only reasonably large study was performed by Kirkpatrick and co-workers, who investigated this hypothesis examining a series of 16 patients with cardiac masses [12]. The highest pixel intensity was found in patients with malignant tumor growth, the lowest in patients with thrombus, and middle pixel intensity was found in patients with benign tumors. The overlap in pixel intensity was rather significant. We did not use echo contrast in this patient since the diagnosis was known and the purpose of the echocardiography examination was to evaluate cardiac function. Most physicians considered transesophageal echocardiography is by the method of choice for optimal visualisation of heart morphology. Transesophageal echocardiography was not performed in our patient because of adequate acoustic windows and because the purpose of the examination was to evaluate cardiac function. Cardiac MRI has in recent years emerged as an excellent tool in diagnosing space-occupying lesions within or in the proximity of the heart [13,14], and has the advantage over CT of not including ionizing radiation or nephrotoxic contrast media. Cardiac MRI could, therefore, play an important role when echocardiography is not conclusive.

There is naturally no consensus or any guidelines for the optimal management of patients with ventricular metastasis of leiomyosarcoma. Surgical resection has been attempted in some cases [4,5], and this could be an option if the tumor growth is restricted in its growth pattern and if the patient is a candidate for surgery. This was considered unrealistic in our patient due to the infiltrative growth of the tumor in the heart combined with multiple metastases.

## Competing interests

The authors declare that they have no competing interests.

## Authors' contributions

MD performed the echocardiography and wrote the manuscript. SV were responsible for the PET/CT. MS was the clinician. All authors approved the final version of the manuscript.

## Supplementary Material

Additional File 1 **Movie 1**. Movie of echocardiography examination from an apical four-chamber view. Note the significant reduction of contractility in the interventricularseptum, presumably due to tumor growth. Click here for file

Additional File 2 **Movie 2**. Movie from a parasternal long-axis view that show tumor engaging the interventricularseptum. Click here for file

Additional File 3 **Movie 3**. Movie from a parasternal short-axis view that show the obliteration of the right ventricular cavity because of tumor growth. Click here for file
